# Supplementary material for: Measuring voluntary responses in healthcare utilization during the COVID-19 pandemic: Evidence from Taiwan
Source: PLoS One. 2022 Dec 8;17(12):e0271810. doi: 10.1371/journal.pone.0271810 (PMC9731448; doi:10.1371/journal.pone.0271810)
Supplement: S1 File — (ZIP) [file pone.0271810.s001.zip › COVID_health_S1_File.pdf]

S1 File. Appendix A: Taiwan's response to the COVID-19 pandemic

Taiwan had been praised by international medias as a success story of COVID-19 prevention [1–6]. As of 9th January 2021, Lowy Institute ranked Taiwan as the top three countries with the best performance on COVID-19 pandemic management [7]. In general, three key strategies have helped the nation successfully prevent the spread of the virus: 1) Early border control and quarantine policies; 2) Distributing and producing face masks and 3) Disclosing COVID-19 information to the public.

Early border control and quarantine policies

As of the first confirmed case, the Taiwan government initiated quarantine policies requiring people returning from "high-risk" COVID-19 countries (e.g., China), and those who had come into contact with confirmed cases, had to enter self-quarantine for 14 days. From March 19th, the Taiwan government restricted all foreigners from entering the country, and on the very same day, all citizens returning from oversea had to take 14 days' quarantine. The border control policy for the foreigners had been relaxed from June 29th. But foreigners entry from other countries need to provide COVID-19 testing with negative results.

Universal use of face masks

In contrast to European and American countries, the Taiwan government considered face masks one of the most important items of personal protective equipment (PPE) for reducing COVID-19 transmission. In order to make sure every resident had access to face masks, at the beginning of the outbreak (i.e. January 24th, 2020) the Taiwan government banned their export and requisitioned a huge increase in local production. The daily production capacity of face mask manufacturers in Taiwan before the outbreak was 1.88 million pieces [8], but currently, Taiwan is able to produce more than 15 million per day [9]. Moreover, starting from February 6th, 2020, the government implemented a name-based rationing system for face masks to curb panic-buying and to ensure the universal face-covering of all residents in Taiwan.

Public disclosure of COVID-19 information

Besides its universal masking policy, border controls and quarantine policies, Taiwan's success in terms of controlling the epidemic can also be attributed to its information dissemination and disclosure strategies. On January 20th, 2020, the Central Epidemic Command Center (CECC) was initiated. When the first confirmed case was corroborated on January 22nd, the CECC held press conferences every day to report on the epidemic and to offer self-protection information to citizens. The frequency of these briefings was reduced to once a week from June 8th, 2020, following a consecutive 8 weeks of no local confirmed cases.

Specifically, the CECC reported newly confirmed cases, cumulative confirmed cases, new death cases and recovered cases every day. The CECC also set up an on-line system for citizens to find out daily data on COVID-19 cases relevant to different counties [10]. In addition, when specific symptoms (such as loss of taste, stroke, etc.) were noted, the CECC also released this information to the public, and whenever any local cases were discovered, it highlighted these during the press conferences with particular emphasis on the source and route of infection. The above information made citizens aware of the severity of the epidemic and helped them monitor their personal health status carefully [11].

Behavioral responses to COVID-19 information

Using Google Trends data, we find that the Taiwanese people responded to the announcement of the first confirmed case immediately by searching for information about the virus and personal protective equipment (PPE), such as face masks and sanitizer. Google trends data is powered by Google, provides the relative search interests of a given keyword made to Google at a given time period and location [12]. Note that instead of showing absolute search volume, Google Trends only provides a relative measure for daily search volume ranging from 0–100, where the numbers represent the search volume relative to the highest point. A value of 100 is the peak popularity of the term, and a value of 50 means half as popular. In order to match the frequency of healthcare data, we aggregate daily data to the weekly level.

Fig A1A suggests that the search intensity of the keywords "Coronavirus" nearly

reached 250 in the week of the first confirmed case announcement. Moreover, this search intensity jumped more than double and reached its peak when the first local COVID-19 case was reported. We also find that PPE-related (i.e. face mask and sanitizer) searches also peaked after the announcement of the first local COVID-19 case (See Fig A1C and A1E). Due to the painful experience of the 2003 outbreak of SARS [13], both the Taiwanese government and its people responded to the first COVID-19 case very quickly indeed [14].

Not every country responded to the first COVID-19 case in such a way, with the United States being a counterexample. Consistent with Bento et al. [15], Fig A1B, A1D and A1F indicates that the information-seeking behavior of the American people was in fact immediate following the first COVID-19 case, which was reported on January 21st, 2020. However, in contrast to Taiwan, the peak of the relative search volume for COVID-19 and PPE-related key words happened on the 7th week (i.e. March 8th to 14th) after the first confirmed case, because the US government verified this case was COVID-19 on March 1st. In addition, the search intensity for face masks (see Fig A1D) peaked after the Center for Disease Control and Prevention (CDC) recommended that people wearing a face mask (April 3rd) would be an effective way to prevent COVID-19 transmission on April 5th to April 11th (i.e. 11 weeks after first confirmed case) [16]. Fig A2 shows the daily trends in search index, The patterns are similar to the weekly trends. Since the United States is a large country, it is possible that people only responded to local cases. In Fig A3, we also find a similar pattern in search behavior, using Google Trends data for Washington State or Seattle, where the first COVID-19 cases happened.

According to a survey conducted by the National Taipei University of Nursing and Health Sciences in April, 97.5% of Taiwanese thought that coronavirus is a serious disease, and over 90% of the interviewees correctly answered questions regarding how the virus spreads and prevention measures [17]. Fig A4 in the Online Appendix shows the percentage of people who say they are wearing face mask when in public places across time, surveyed by YouGov [18]. The figure shows that as early as in February, over 80% of Taiwanese said that they were wearing a face mask in a public space. Further, the portion of people wearing masks remains high through the time. In contrast, only 7% of Americans said they had worn a face mask in early March, and

Fig A1. Google search intensity for COVID-19 related keywords: Taiwan and US (weekly). A: Coronavirus, Taiwan. B: Coronavirus, US. C: Mask, Taiwan. D: Mask, US. E: Sanitizer, Taiwan. F: Sanitizer, US. The figures are constructed by using Google Trends data. Google Trends only provides a relative measure for daily search volume ranging from 0–100. In order to match the frequency of healthcare data, we aggregate daily data to the weekly level. For Taiwan's keywords, we use the equivalent term in Chinese for Coronavirus, mask, and sanitizer as the keywords.

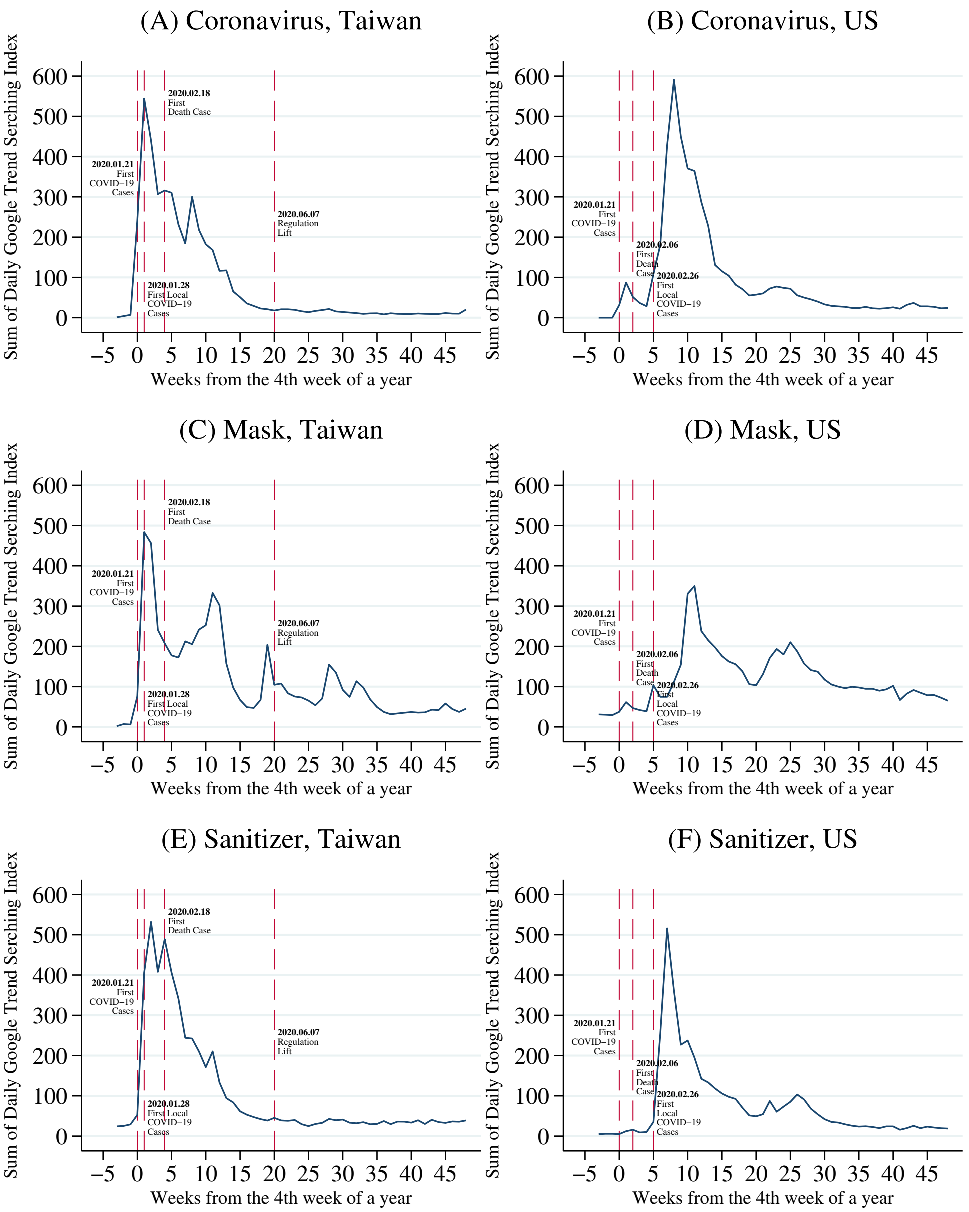

the proportion began raised to half only until mid-April. All of these results are consistent with the patterns seen in Google Trends data, suggesting that the

Fig A2. Google search intensity for COVID-19 related keywords: Taiwan and US (daily). A: Coronavirus, Taiwan. B: Coronavirus, US. C: Mask, Taiwan. D: Mask, US. E: Sanitizer, Taiwan. F: Sanitizer, US. The figures are constructed by using Google Trends data. Google Trends only provides a relative measure for daily search volume ranging from 0–100. In order to match the frequency of healthcare data, we aggregate daily data to the weekly level. For Taiwan's keywords, we use the equivalent term in Chinese for Coronavirus, mask, and sanitizer as the keywords.

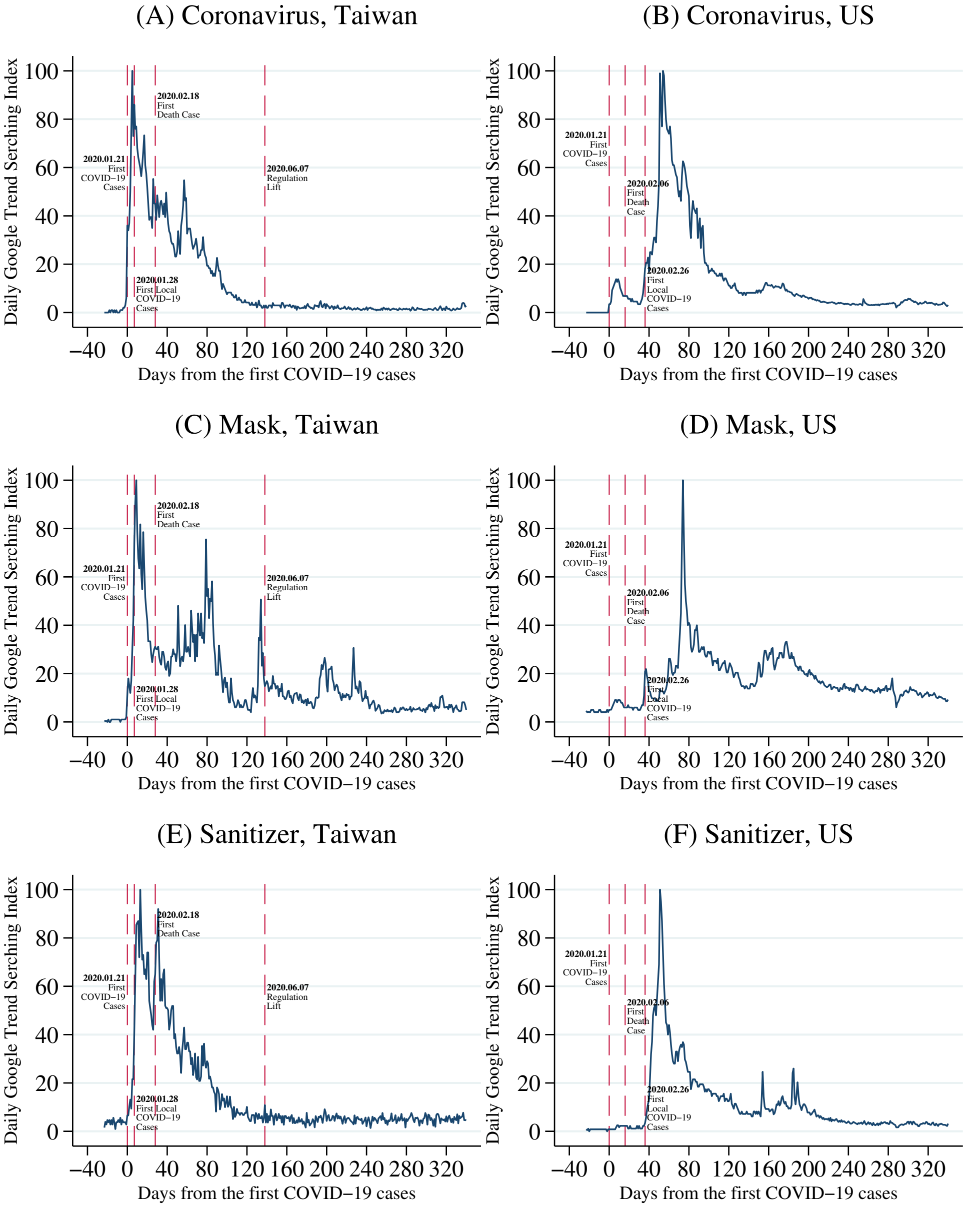

Taiwanese people have responded to COVID-19 in a rapid and proactive way.

Fig A3. Google search intensity for COVID-19 related keywords: Washington and Seattle (weekly). A: Coronavirus, Washington. B: Coronavirus, Seattle. C: Mask, Washington. D: Mask, Seattle. E: Sanitizer, Washington. F: Sanitizer, Seattle. The figures are constructed by using Google Trends data. The daily search volume ranging from 0–100. For Taiwan's keywords, we use the equivalent term in Chinese for Coronavirus, mask, and sanitizer as the keywords.

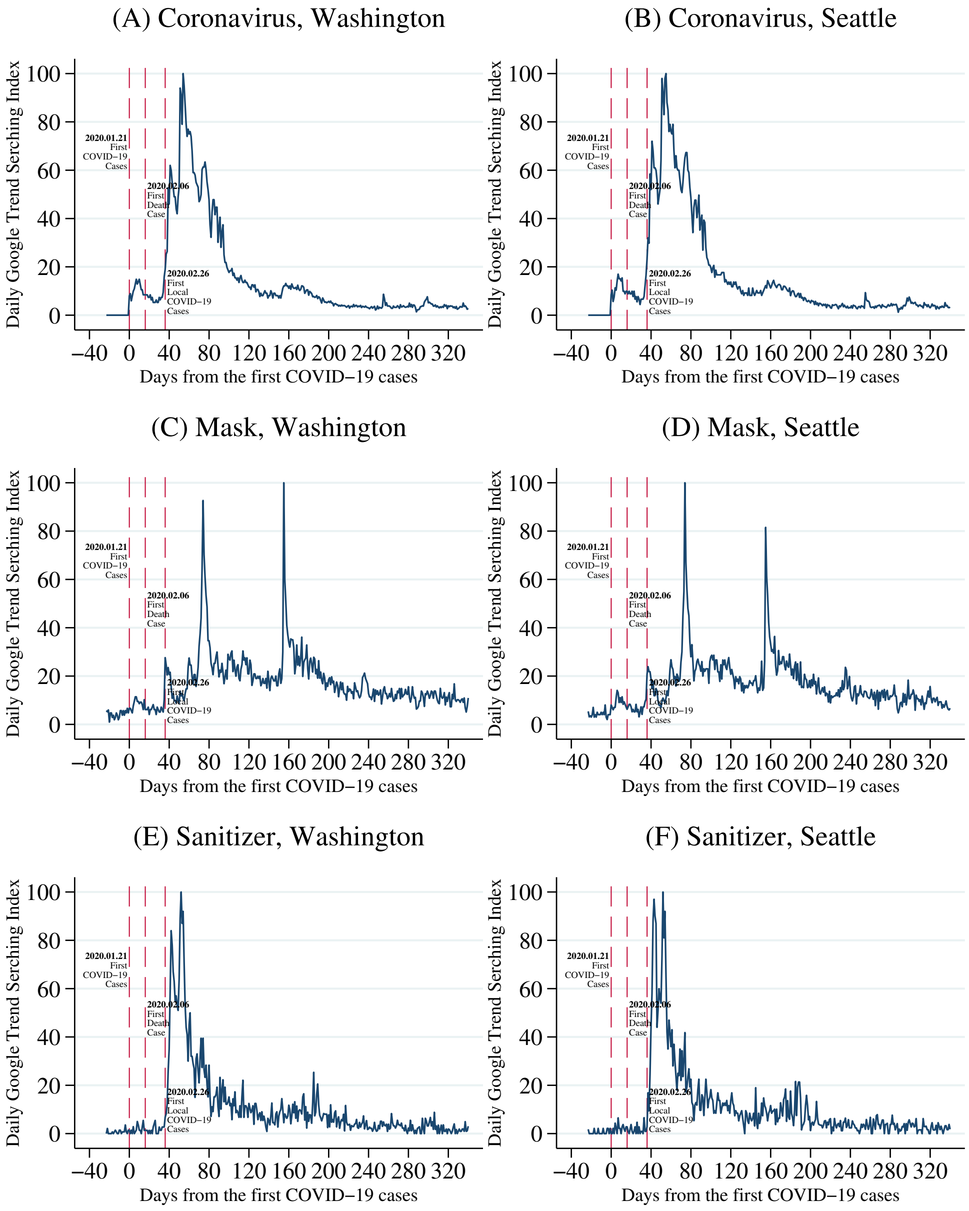

Fig A4. Share of people wearing a face mask when in public space. Data source is from YouGov [18].

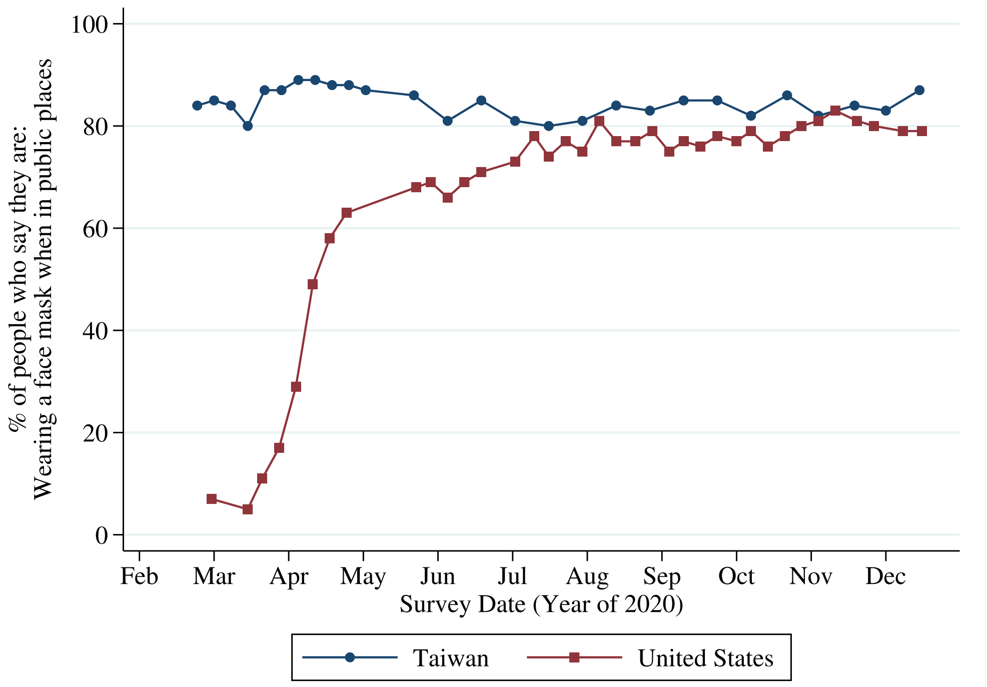

References

1. Taiwan celebrates equality, coronavirus success in Asia's largest pride march. Reuters [Internet] 2020 Oct 31. [cited 2022 Mar 24];. Available from: https://www.reuters.com/article/uk-gay-pride-taiwan-idUKKBN27G0CM.

2. Covid? What Covid? Taiwan Thrives as a bubble of normality. The New York Times [Internet] 2021 Mar 13. [cited 2022 Mar 24];. Available from: https://www.nytimes.com/2021/03/13/world/asia/taiwan-covid.html.

3. Taiwan, a Covid-19 outlier, is selling something scarce: life without fear of the virus. The New York Times [Internet] 2021 Mar 13. [cited 2022 Mar 24];. Available from: https://www.nytimes.com/2021/03/13/world/taiwan-a-covid-19-outlier-is-selling-something-scarce-life-without-fear-o[illegible] html.

4. Taiwan just went 200 days without a locally transmitted Covid-19 case. Here's how they did it. CNN [Internet] 2020 Oct 29. [cited 2022 Mar 24]; 2020. Available from: https://www.cnn.com/2020/10/29/asia/taiwan-covid-19-intl-hnk.

5. Taiwan's coronavirus response is among the best globally. CNN [Internet] 2020 Dec 2. [cited 2022 Mar 24];. Available from: https://www.cnn.com/2020/04/04/asia/taiwan-coronavirus-response-who-intl-hnk/index.html.

6. Covid-19 has ravaged economies all over the world—but not Taiwan's. Economist [Internet] 2020 Dec 2. [cited 2022 Mar 24];. Available from: https://www.economist.com/asia/2020/12/02/covid-19-has-ravaged-economies-all-over-the-world-but-not-taiwans.

7. Lowy Institute. Covid Performance Index [Internet]. 2021 [updated 2021 Mar 13; cited 2022 Mar 24];. Available from: https://interactives.lowyinstitute.org/features/covid-performance/.

8. Ministry of Economic Affairs. The production and supply of masks are secured, please do not need to over panic and hoard masks [Internet]. 2020 [updated

2020 Jan 22; cited 2022 Mar 24];. Available from: https://www.moea.gov.tw/MNS/populace/news/News.aspx?kind=1&menu_id=40&news_id=88545.

9. Ministry of Economic Affairs. The daily production capacity of masks is 15 million pieces is the production goal, not the production limit [Internet]. 2020 [updated 2020 Apr 10; cited 2022 Mar 24];. Available from: https://www.moea.gov.tw/MNS/populace/news/News.aspx?kind=9&menu_id=22333&news_id=89290.

10. Taiwan Centers for Disease Control. Infectious disease statistics query system [Internet]. 2022 [updated 2022 Mar 24; cited 2022 Mar 24];. Available from: https://nidss.cdc.gov.tw/Home/Index?aspxerrorpath=/ch/NIDSS/_DiseaseMap.aspx.

11. Wang YH. Development trend of convergence of communication and communication industry investigation and analysis—Radio and TV market survey report. National Communications Commission Organization; 2020.

12. Google. Google trends [Internet]. 2022 [updated 2022 Mar 24; cited 2022 Mar 24];. Available from: https://trends.google.com.tw/.

13. Bennett D, Chiang CF, Malani A. Learning during a crisis: The SARS epidemic in Taiwan. Journal of Development Economics. 2015;112:1–18.

14. Chen KT, Twu SJ, Chang HL, Wu YC, Chen CT, Lin TH, et al. SARS in Taiwan: an overview and lessons learned. International Journal of Infectious Diseases. 2005;9(2):77–85.

15. Bento AI, Nguyen T, Wing C, Lozano-Rojas F, Ahn YY, Simon K. Evidence from internet search data shows information-seeking responses to news of local COVID-19 cases. Proceedings of the National Academy of Sciences. 2020;117(21):11220–2.

16. Geggel L. Everyone should wear face 'masks' in public, CDC now recommends. Live Science [Internet]. 2020;.

17. National Taipei University of Nursing and Health Sciences released a poll comparing the SARS and Covid-19 epidemics. Taiwan Hot [Internet];.

18. Smith M. International COVID-19 tracker update: 18 May. National Communications Commission Organization; 2020.
