# Supplementary material for: Measuring voluntary responses in healthcare utilization during the COVID-19 pandemic: Evidence from Taiwan
Source: PLoS One. 2022 Dec 8;17(12):e0271810. doi: 10.1371/journal.pone.0271810 (PMC9731448; doi:10.1371/journal.pone.0271810)

S2 Appendix. Additional results

Table B1. Poisson estimated coefficient (DID design)

	(1)	(2)	(3)	(4)	(5)	(6)	(7)	(8)
	Outpatient Care				Inpatient Care			
**Panel A:** All diseases								
$Y_{2020} \times Post$	–0.14***	–0.13***	–0.14***	–0.14***	–0.05***	–0.04**	–0.05	–0.04
	(0.01)	(0.02)	(0.02)	(0.02)	(0.00)	(0.01)	(0.03)	(0.03)
**Panel B:** ILI diseases								
$Y_{2020} \times Post$	–0.46***	–0.47***	–0.47***	–0.48***	–0.51***	–0.52***	–0.51***	–0.50***
	(0.04)	(0.04)	(0.04)	(0.04)	(0.02)	(0.05)	(0.07)	(0.06)
**Panel C:** Non-ILI diseases								
$Y_{2020} \times Post$	–0.10***	–0.10***	–0.11***	–0.11***	–0.02***	–0.01	–0.02	–0.02
	(0.00)	(0.02)	(0.02)	(0.02)	(0.00)	(0.02)	(0.03)	(0.03)
Observation	8,008							
Basic control	✓	✓	✓	✓	✓	✓	✓	✓
Demographic variables		✓	✓			✓	✓	
Weather variables		✓	✓	✓		✓	✓	✓
County fixed effect			✓	✓			✓	✓
County-by-year fixed effect				✓				✓
County-by-week fixed effect				✓				✓
County specific time trend				✓				✓

Note: This table shows the estimated γ_0 (i.e. the coefficient on $Year_{2020} \times Post_d$) in the equation (1), which is a Poisson regression. Sample period is 2014–2020. *Basic Control* includes the year fixed effect, the week fixed effect and various holiday dummies such as, New Year Eve, New Year, Lunar New Year, Peace Memorial Day, Qing-Ming Festival, Labor's Day, and Dragon Boat Festival, Moon Festival, and National Day. *Demographic variables* includes annually county-level age structure, sex ratio, educational attainment. *Weather variables* includes weekly county-level temperatures and precipitation. All regressions are weighted by the monthly population size of a county. Robust standard errors clustered at the year-week and county levels are reported in parentheses.
$^{*}p < 0.05$ $^{**}p < 0.01$ $^{***}p < 0.001$

Table B2. Poisson estimated coefficient (multi-period DID design)

	(1)	(2)	(3)	(4)	(5)	(6)	(7)	(8)
	Outpatient Care				Inpatient Care			
**Panel A:** All diseases								
$Y_{2020} \times Pandemic$	–0.21***	–0.19***	–0.21***	–0.21***	–0.12***	–0.10***	–0.12***	–0.11***
	(0.01)	(0.02)	(0.02)	(0.02)	(0.00)	(0.02)	(0.03)	(0.03)
$Y_{2020} \times CovidFree$	–0.09***	–0.08***	–0.09***	–0.09***	–0.01**	0.00	–0.01	–0.00
	(0.01)	(0.02)	(0.02)	(0.02)	(0.00)	(0.01)	(0.03)	(0.03)
**Panel B:** ILI diseases								
$Y_{2020} \times Pandemic$	–0.55***	–0.56***	–0.57***	–0.57***	–0.53***	–0.54***	–0.53***	–0.52***
	(0.06)	(0.06)	(0.06)	(0.06)	(0.05)	(0.06)	(0.08)	(0.07)
$Y_{2020} \times CovidFree$	–0.38***	–0.39***	–0.40***	–0.40***	–0.50***	–0.50***	–0.49***	–0.49***
	(0.03)	(0.03)	(0.04)	(0.04)	(0.02)	(0.05)	(0.07)	(0.06)
**Panel C:** Non-ILI diseases								
$Y_{2020} \times Pandemic$	–0.17***	–0.16***	–0.17***	–0.17***	–0.09***	–0.07***	–0.09**	–0.08**
	(0.01)	(0.02)	(0.02)	(0.02)	(0.00)	(0.02)	(0.03)	(0.03)
$Y_{2020} \times CovidFree$	–0.06***	–0.06**	–0.07***	–0.07***	0.02***	0.03	0.02	0.02
	(0.01)	(0.02)	(0.02)	(0.02)	(0.00)	(0.02)	(0.03)	(0.03)
Observation	8,008							
Basic control	✓	✓	✓	✓	✓	✓	✓	✓
Demographic variables		✓	✓			✓	✓	
Weather variables		✓	✓	✓		✓	✓	✓
County fixed effect			✓	✓			✓	✓
County-by-year fixed effect				✓				✓
County-by-week fixed effect				✓				✓
County specific time trend				✓				✓

Note: This table shows the estimated γ_1 (i.e. the coefficient on $Year_{2020} \times Pandemic_d$) and γ_2 (i.e. the coefficient on $Year_{2020} \times CovidFree_d$) in the equation (2), which is a Poisson regression. Sample period is 2014–2020. *Basic Control* includes the year fixed effect, the week fixed effect and various holiday dummies such as, New Year Eve, New Year, Lunar New Year, Peace Memorial Day, Qing-Ming Festival, Labor's Day, and Dragon Boat Festival, Moon Festival, and National Day. *Demographic variables* includes annually county-level age structure, sex ratio, educational attainment. *Weather variables* includes weekly county-level temperatures and precipitation. All regressions are weighted by the monthly population size of a county. Robust standard errors clustered at the year-week and county levels are reported in parentheses.
$^{*}p < 0.05$ $^{**}p < 0.01$ $^{***}p < 0.001$

Table B3. Robustness check: Clustering levels of standard errors (DID design)

	(1)	(2)	(3)	(4)	(5)	(6)	(7)	(8)
	Outpatient Care				Inpatient Care			
**Panel A:** All diseases								
$Y_{2020} \times Post$	0.87	0.88	0.87	0.87	0.95	0.96	0.95	0.96
Cluster at county level	[0.86,0.89]	[0.87,0.89]	[0.86,0.88]	[0.86,0.88]	[0.90,1.00]	[0.91,1.01]	[0.91,1.00]	[0.91,1.01]
Cluster at year-week level	[0.84,0.91]	[0.84,0.92]	[0.87,0.87]	[0.84,0.90]	[0.91,0.99]	[0.92,1.01]	[0.91,0.99]	[0.92,1.00]
**Panel B:** ILI diseases								
$Y_{2020} \times Post$	0.63	0.63	0.62	0.62	0.60	0.60	0.60	0.60
Cluster at county level	[0.62,0.65]	[0.61,0.65]	[0.61,0.64]	[0.60,0.64]	[0.54,0.66]	[0.54,0.66]	[0.54,0.66]	[0.55,0.67]
Cluster at year-week level	[0.58,0.69]	[0.58,0.68]	[0.62,0.62]	[0.57,0.67]	[0.55,0.66]	[0.54,0.65]	[0.55,0.66]	[0.55,0.66]
**Panel C:** Non-ILI diseases								
$Y_{2020} \times Post$	0.90	0.91	0.90	0.90	0.98	0.99	0.98	0.98
Cluster at county level	[0.89,0.91]	[0.90,0.92]	[0.89,0.91]	[0.89,0.91]	[0.92,1.03]	[0.94,1.05]	[0.93,1.03]	[0.93,1.04]
Cluster at year-week level	[0.86,0.94]	[0.87,0.95]	[0.87,0.93]	[0.87,0.93]	[0.93,1.02]	[0.94,1.04]	[0.94,1.02]	[0.94,1.02]
Observation	8,008							
Basic control	✓	✓	✓	✓	✓	✓	✓	✓
Demographic variables		✓	✓			✓	✓	
Weather variables		✓	✓	✓		✓	✓	✓
County fixed effect			✓	✓			✓	✓
County-by-year fixed effect				✓				✓
County-by-week fixed effect				✓				✓
County specific time trend				✓				✓

Note: This table shows the incidence-rate ratios (IRR) for the estimated γ_0 (i.e. the coefficient on $Year_{2020} \times Post_d$) in the equation (1), which is a Poisson regression. Sample period is 2014–2020. *Basic Control* includes the year fixed effect, the week fixed effect and various holiday dummies such as, New Year Eve, New Year, Lunar New Year, Peace Memorial Day, Qing-Ming Festival, Labor's Day, and Dragon Boat Festival, Moon Festival, and National Day. *Demographic variables* includes annually county-level age structure, sex ratio, educational attainment. *Weather variables* includes weekly county-level temperatures and precipitation. All regressions are weighted by the monthly population size of a county. Robust standard errors clustered at the year-week and county levels. 95% CI computed reported in squared brackets.

Table B4. Robustness check: Clustering levels of standard errors (multi-period DID design)

	(1)	(2)	(3)	(4)	(5)	(6)	(7)	(8)
	Outpatient Care				Inpatient Care			
**Panel A:** All diseases								
$Y_{2020} \times Pandemic$	0.81	0.82	0.81	0.81	0.89	0.90	0.89	0.90
Cluster at county level	[0.80,0.83]	[0.81,0.84]	[0.80,0.83]	[0.80,0.83]	[0.84,0.93]	[0.86,0.95]	[0.85,0.93]	[0.85,0.94]
Cluster at year-week level	[0.78,0.85]	[0.78,0.87]	[0.78,0.85]	[0.78,0.85]	[0.84,0.93]	[0.86,0.95]	[0.85,0.93]	[0.85,0.94]
$Y_{2020} \times CovidFree$	0.92	0.92	0.91	0.91	0.99	1.00	0.99	1.00
Cluster at county level	[0.90,0.93]	[0.91,0.93]	[0.90,0.92]	[0.90,0.92]	[0.94,1.05]	[0.95,1.05]	[0.94,1.05]	[0.94,1.05]
Cluster at year-week level	[0.88,0.95]	[0.88,0.96]	[0.88,0.94]	[0.88,0.94]	[0.95,1.03]	[0.96,1.05]	[0.95,1.03]	[0.96,1.04]
**Panel B:** ILI diseases								
$Y_{2020} \times Pandemic$	0.57	0.57	0.57	0.56	0.59	0.58	0.59	0.59
Cluster at county level	[0.56,0.59]	[0.55,0.59]	[0.55,0.58]	[0.55,0.58]	[0.52,0.66]	[0.52,0.65]	[0.52,0.66]	[0.53,0.66]
Cluster at year-week level	[0.51,0.65]	[0.51,0.64]	[0.57,0.57]	[0.50,0.64]	[0.52,0.66]	[0.52,0.66]	[0.52,0.66]	[0.53,0.67]
$Y_{2020} \times CovidFree$	0.68	0.68	0.67	0.67	0.61	0.60	0.61	0.61
Cluster at county level	[0.66,0.70]	[0.65,0.70]	[0.65,0.69]	[0.65,0.69]	[0.55,0.67]	[0.55,0.67]	[0.55,0.67]	[0.56,0.67]
Cluster at year-week level	[0.64,0.73]	[0.63,0.72]	[0.67,0.67]	[0.62,0.71]	[0.55,0.67]	[0.55,0.67]	[0.55,0.67]	[0.55,0.67]
**Panel C:** Non-ILI diseases								
$Y_{2020} \times Pandemic$	0.84	0.86	0.85	0.85	0.91	0.93	0.92	0.92
Cluster at county level	[0.83,0.86]	[0.84,0.87]	[0.83,0.86]	[0.83,0.86]	[0.86,0.96]	[0.88,0.98]	[0.87,0.96]	[0.87,0.97]
Cluster at year-week level	[0.81,0.88]	[0.81,0.90]	[0.81,0.88]	[0.81,0.88]	[0.87,0.96]	[0.88,0.98]	[0.87,0.96]	[0.88,0.96]
$Y_{2020} \times CovidFree$	0.94	0.95	0.94	0.94	1.02	1.03	1.02	1.02
Cluster at county level	[0.93,0.95]	[0.93,0.96]	[0.92,0.95]	[0.92,0.95]	[0.96,1.08]	[0.97,1.09]	[0.96,1.08]	[0.97,1.08]
Cluster at year-week level	[0.90,0.98]	[0.90,0.99]	[0.90,0.97]	[0.90,0.97]	[0.98,1.06]	[0.98,1.08]	[0.98,1.06]	[0.98,1.06]
Observation	8,008							
Basic control	✓	✓	✓	✓	✓	✓	✓	✓
Demographic variables		✓	✓			✓	✓	
Weather variables		✓	✓	✓		✓	✓	✓
County fixed effect			✓	✓			✓	✓
County-by-year fixed effect				✓				✓
County-by-week fixed effect				✓				✓
County specific time trend				✓				✓

Note: This table shows the incidence-rate ratios (IRR) for the estimated γ_1 (i.e. the coefficient on $Year_{2020} \times Pandemic_d$) and γ_2 (i.e. the coefficient on $Year_{2020} \times CovidFree_d$) in the equation (2), which is a Poisson regression. Sample period is 2014–2020. *Basic Control* includes the year fixed effect, the week fixed effect and various holiday dummies such as, New Year Eve, New Year, Lunar New Year, Peace Memorial Day, Qing-Ming Festival, Labor's Day, and Dragon Boat Festival, Moon Festival, and National Day. *Demographic variables* includes annually county-level age structure, sex ratio, educational attainment. *Weather variables* includes weekly county-level temperatures and precipitation. All regressions are weighted by the monthly population size of a county. Robust standard errors clustered at the year-week and county levels. 95% CI reported in squared brackets.

Table B5. Robustness check: Unweighted regressions (DID design)

	(1)	(2)	(3)	(4)	(5)	(6)	(7)	(8)
	Outpatient Care				Inpatient Care			
**Panel A:** All diseases								
$Y_{2020} \times Post$	0.88***	0.89***	0.88***	0.87***	0.95***	0.96	0.95	0.95
	[0.87,0.89]	[0.85,0.94]	[0.84,0.91]	[0.84,0.91]	[0.94,0.96]	[0.91,1.01]	[0.89,1.01]	[0.90,1.01]
**Panel B:** ILI diseases								
$Y_{2020} \times Post$	0.65***	0.65***	0.64***	0.64***	0.57***	0.57***	0.57***	0.57***
	[0.61,0.70]	[0.60,0.70]	[0.59,0.69]	[0.59,0.69]	[0.48,0.68]	[0.48,0.68]	[0.46,0.70]	[0.46,0.70]
**Panel C:** Non-ILI diseases								
$Y_{2020} \times Post$	0.91***	0.92***	0.90***	0.90***	0.98***	0.99	0.98	0.99
	[0.90,0.91]	[0.88,0.96]	[0.87,0.94]	[0.87,0.94]	[0.98,0.98]	[0.93,1.04]	[0.93,1.04]	[0.93,1.04]
Observation	8,008							
Basic control	✓	✓	✓	✓	✓	✓	✓	✓
Demographic variables		✓	✓			✓	✓	
Weather variables		✓	✓	✓		✓	✓	✓
County fixed effect			✓	✓			✓	✓
County-by-year fixed effect				✓				✓
County-by-week fixed effect				✓				✓
County specific time trend				✓				✓

Note: This table shows the incidence-rate ratios (IRR) for the estimated γ_0 (i.e. the coefficient on $Year_{2020} \times Post_d$) in the equation (1), which is a Poisson regression. Sample period is 2014–2020. *Basic Control* includes the year fixed effect, the week fixed effect and various holiday dummies such as, New Year Eve, New Year, Lunar New Year, Peace Memorial Day, Qing-Ming Festival, Labor's Day, and Dragon Boat Festival, Moon Festival, and National Day. *Demographic variables* includes annually county-level age structure, sex ratio, educational attainment. *Weather variables* includes weekly county-level temperatures and precipitation. Robust standard errors clustered at the year-week and county levels. 95% CI reported in squared brackets.

$^{*}p < 0.05$ $^{**}p < 0.01$ $^{***}p < 0.001$

Table B6. Robustness check: Unweighted regressions (multi-period DID Design)

	(1)	(2)	(3)	(4)	(5)	(6)	(7)	(8)
	Outpatient Care				Inpatient Care			
**Panel A:** All diseases								
$Y_{2020} \times Pandemic$	0.83***	0.84***	0.82***	0.82***	0.89***	0.90***	0.90***	0.90***
	[0.82,0.84]	[0.80,0.89]	[0.79,0.86]	[0.79,0.86]	[0.89,0.90]	[0.85,0.95]	[0.84,0.95]	[0.85,0.96]
$Y_{2020} \times CovidFree$	0.92***	0.93**	0.91***	0.91***	0.99***	0.99	0.99	0.99
	[0.91,0.93]	[0.89,0.97]	[0.88,0.95]	[0.88,0.95]	[0.98,0.99]	[0.94,1.05]	[0.93,1.05]	[0.93,1.05]
**Panel B:** ILI diseases								
$Y_{2020} \times Pandemic$	0.59***	0.59***	0.58***	0.58***	0.54***	0.54***	0.54***	0.54***
	[0.53,0.66]	[0.52,0.66]	[0.51,0.65]	[0.51,0.65]	[0.45,0.65]	[0.44,0.66]	[0.43,0.67]	[0.43,0.68]
$Y_{2020} \times CovidFree$	0.71***	0.70***	0.69***	0.69***	0.59***	0.60***	0.59***	0.59***
	[0.67,0.74]	[0.66,0.75]	[0.65,0.74]	[0.64,0.74]	[0.50,0.71]	[0.50,0.72]	[0.48,0.73]	[0.47,0.73]
**Panel C:** Non-ILI diseases								
$Y_{2020} \times Pandemic$	0.86***	0.88***	0.86***	0.86***	0.93***	0.93*	0.93*	0.93*
	[0.85,0.86]	[0.84,0.92]	[0.82,0.89]	[0.82,0.89]	[0.92,0.93]	[0.88,0.99]	[0.88,0.99]	[0.88,0.99]
$Y_{2020} \times CovidFree$	0.94***	0.96*	0.94***	0.94***	1.02***	1.02	1.02	1.02
	[0.93,0.95]	[0.92,1.00]	[0.90,0.97]	[0.90,0.97]	[1.01,1.02]	[0.97,1.08]	[0.96,1.08]	[0.96,1.08]
Observation	8,008							
Basic control	✓	✓	✓	✓	✓	✓	✓	✓
Demographic variables		✓	✓			✓	✓	
Weather variables		✓	✓	✓		✓	✓	✓
County fixed effect			✓	✓			✓	✓
County-by-year fixed effect				✓				✓
County-by-week fixed effect				✓				✓
County specific time trend				✓				✓

Note: This table shows the incidence-rate ratios (IRR) for the estimated γ_1 (i.e. the coefficient on $Year_{2020} \times Pandemic_d$) and γ_2 (i.e. the coefficient on $Year_{2020} \times CovidFree_d$) in the equation (2), which is a Poisson regression. Sample period is 2014–2020. *Basic Control* includes the year fixed effect, the week fixed effect and various holiday dummies such as, New Year Eve, New Year, Lunar New Year, Peace Memorial Day, Qing-Ming Festival, Labor's Day, and Dragon Boat Festival, Moon Festival, and National Day. *Demographic variables* includes annually county-level age structure, sex ratio, educational attainment. *Weather variables* includes weekly county-level temperatures and precipitation. Robust standard errors clustered at the year-week and county levels. 95% CI reported in squared brackets.
$^{*}p < 0.05$ $^{**}p < 0.01$ $^{***}p < 0.001$

Fig B1. Dynamic effects of COVID-19 outbreak on non-COVID-19 health utilization (Poisson estimated coefficient). A: Outpatient care: ILI diseases. B: Outpatient care: Non-ILI diseases. C: Inpatient care: ILI diseases. D: Inpatient care: Non-ILI diseases. This figure shows the estimated β_d in equation (3). The dashed lines represent the corresponding 95% confidence intervals. The horizontal axis denotes the number of weeks from the COVID-19 outbreak (i.e., the 4th week in a year). The top (bottom) panel displays the results for outpatient (inpatient) care by disease type. Sample size is 8,008 and sample period is 2014–2020.

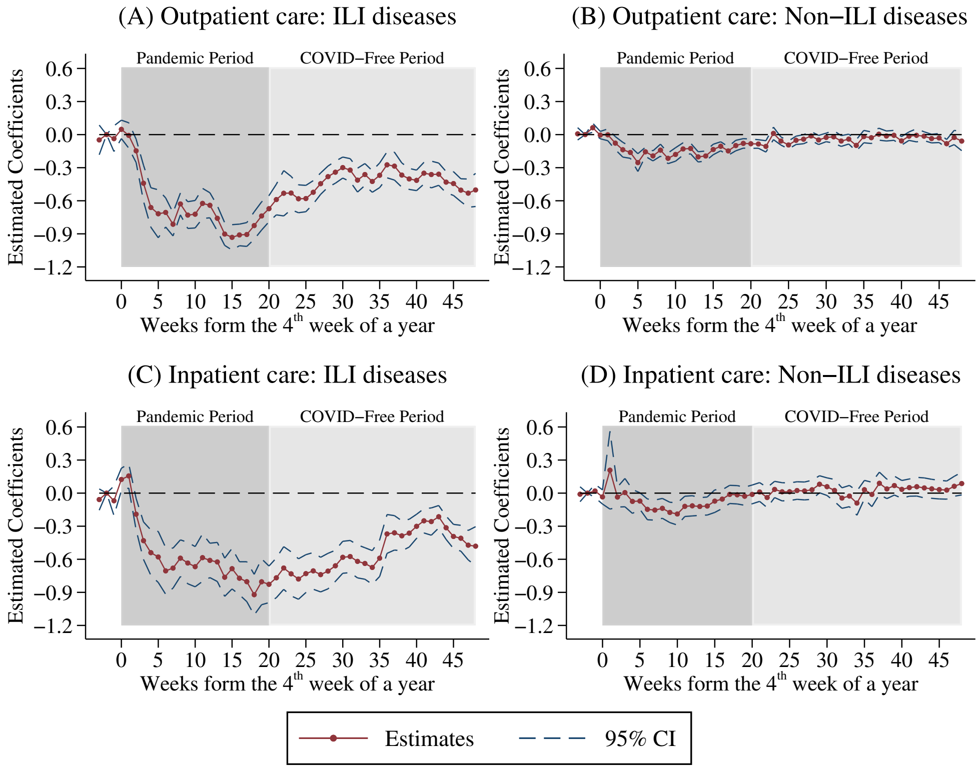

Supplement: S2 File — (ZIP) [file pone.0271810.s002.zip › COVID_health_S2_File.pdf]
