# Supplementary material for: Measuring voluntary responses in healthcare utilization during the COVID-19 pandemic: Evidence from Taiwan
Source: PLoS One. 2022 Dec 8;17(12):e0271810. doi: 10.1371/journal.pone.0271810 (PMC9731448; doi:10.1371/journal.pone.0271810)
Supplement: Attachment — Submitted filename: COVID_health_PLOS_referee2_v2_YungYu.pdf [file pone.0271810.s003.pdf]

Authors' Response to Referee 2's Comments

Dear Referee:

Thank you very much for your insightful suggestions, all of which have helped us improve the manuscript significantly. In response to your comments, we have made the following revisions. Please let us know if you have any further questions.

Referee 2's Comment:

1. Please do review the manuscript organization to align with Plos ONE guidelines (see `https://journals.plos.org/plosone/s/submission-guidelines`)

- Note Abstract/Introduction/Materials and methods/Results/Discussion/Conclusions: All these pieces are present in the manuscript but they are not in that sequence.

- For the readers of Plos ONE it is too much to have such an Introduction + Background extension (6 pages and something more). Please just make on Background and do not put results or discuss your results on it. End this section with the aims.

Authors' Response:

• Thank you for your advice. We have re-formatted our manuscript by following the PLoS One submission guidelines. We have also adjusted our manuscript, and it now has six sections: Abstract, Introduction, Materials and methods, Results, Discussion, and Conclusion. Specifically, we have moved the original Background section into S1 Appendix A and integrated some information into the revised Introduction section as follows:

From the 42nd line on page 4:

"The case of Taiwan is well-suited for this analysis—for three important reasons. First, as of the end of 2020, Taiwan had only experienced seven deaths and 799 COVID-19 cases. The total population of Taiwan is around 23 million, so the country's infection rate is very low. Moreover, no severe local transmissions had occurred in 2020. Fig 1 displays the number of new confirmed cases for each week of 2020. The first confirmed COVID-19 case was announced in the 4th week of 2020 (i.e., the week of January 21st). The dark color represents the number of new local confirmed cases in a given week, and the gray color means the weekly number of new non-local cases. Fewer than 10% of confirmed cases

are local ones, and there have been no new confirmed local cases from the 17th to 51st week of 2020 (i.e., April 12th to December 22nd, 2020). So the pandemic had a very limited impact on healthcare capacity in the country, thereby helping us rule out unmet demand due to supply-side factors."

Fig 1. Weekly number of newly confirmed COVID-19 cases in Taiwan. This figure displays weekly numbers of newly confirmed COVID-19 cases in Taiwan. The dark color represents the number of new local confirmed cases in a given week, and the gray color means weekly numbers of new imported cases. Data source comes from TCDC.

- We have removed the results discussion section from the Introduction.
- We have added one paragraph at the end of the Introduction section in order to highlight the aims of the study:

From the 97th line on page 6:

"The primary aim of this paper is to examine the voluntary response in health demand during the COVID-19 outbreak. In addition, this paper also utilizes the unique "COVID-free" period in Taiwan during the second half of 2020 to examine whether and to what extent the spontaneous response persists in the post-pandemic period. Furthermore, this paper compares the heterogeneous impact of outpatient and inpatient care as well as the ILI and non-ILI diseases to decompose the potential mechanisms of the pandemic's impact on health demand responses."

Referee 2's Comment:

2. The same issue of formatting with citations. Please see the guidelines. PLOS uses "Vancouver" style.

Authors' Response:

- Thank you for reminding us of this. We have re-formatted our manuscript according to the PLoS One submission guidelines. In addition to the citations format, we have also revised the formats for the section titles, tables, figures, captions, and footnotes, based on the guidelines.

Referee 2's Comment:

3. For the models, the authors state that they used the Poisson regression and a multiway approach to

compute standard errors clustered both at the year-week and county level. Does this address potential overdispersion so common in count data?

Authors' Response:

• Thank you for raising this concern. Initially, we adopted the multiway clustering approach to deal with the potential correlation of error terms at the year-week and county levels. However, we found that this approach can also mitigate concern about overdispersion, so we have restated the relevant statements as follows:

From the 188th line on page 9:

"Throughout our analysis, we use the multiway clustering approach proposed by Cameron, Gelbach, and Miller [59] to calculate standard errors clustered at both the year-week and the county levels. We adopt the multiway clustered standard error due to two concerns. First, there are potential within-group correlations of errors associated with both the county cluster and the year-week cluster. We apply the clustered standard error approach to account for this correlation pattern. Secondly, there is an overdispersion issue in our outcome variables. As shown in Table 1, the standard deviations of the outpatient visits/inpatient admissions are far exceed the squared root of the mean. Overdispersion problems in count data are sometimes caused by clustering [60]. We observe in our data that any variances in outcomes variables within the county cluster and the year-week cluster are smaller than the variances for the whole sample. Therefore, we apply the multiway clustered standard error to account for the overdispersion problems due to clustering. Finally, we conduct robustness checks on the estimates by computing the standard errors at different cluster levels."

Referee 2's Comment:

4. Also, the authors indicate to have applied weighted regression by population size of a county. Why this weighting?

Authors' Response:

• Thank you for raising this issue. In the revised manuscript, we have discussed why we applied weighted regression by the population size of a county. We restate the relevant statements as follows:

From the 203rd line on page 10:

"Finally, all regressions are weighted by the monthly population size of a county. As shown in Table 1, a fairly variation in population size across counties exists. Specifically, the population size ranges from 12 thousand to 4 million. To obtain estimates that represent the population average, we weighted all of our estimations with population size. In a latter section, we also report the results on unweighted regressions as a robustness check."

Referee 2's Comment:

5. Please document the software used, and if possible share the code and the data used for this analysis. This is a quite pedagogic work.

Authors' Response:

• Thank you for reminding us of this matter. In the revised manuscript, we have added this information and restate the relevant information as follows:

From the 186th line on page 10:

"We perform our analysis with Stata 16 through the package *ppmlhdfe* [58]."

• We have also published our data and code online and added a data and code availability summary on page 22. Information on the section is as follows:

From the 505th line on page 23:

"The data and codes used in this paper are available at:
`https://github.com/yungyutsai/Taiwan_COVID19_Healthcare_Utilization`."

Referee 2's Comment:

6. I could not see the figure 3.

Authors' Response:

• Thank you for reminding us of this. We have followed the PLoS ONE submission guideline to reformat all of our figures. Specifically, we have uploaded the figure files separately and only include figure captions in the main manuscript. All figures are now included in the **COVID_health_Figures_File_v3.pdf file.** Figure 3 is as follows. Note that we have followed

your comment 8 and reported on the exponentiated version of results (i.e., incidence rate ratios [IRR]) instead.

Fig 3. Dynamic effects of the COVID-19 outbreak on non-COVID-19 health utilization. A: Outpatient care: ILI diseases. B: Outpatient care: Non-ILI diseases. C: Inpatient care: ILI diseases. D: Inpatient care: Non-ILI diseases. This figure shows the IRR of the estimated β_d in equation (3). The dashed lines represent the corresponding 95% confidence intervals. The horizontal axis denotes the number of weeks from the COVID-19 outbreak (i.e., the 4th week in a year). The top (bottom) panel displays the results for outpatient (inpatient) care by disease type. Sample size is 8,008, and the sample period is 2014–2020.

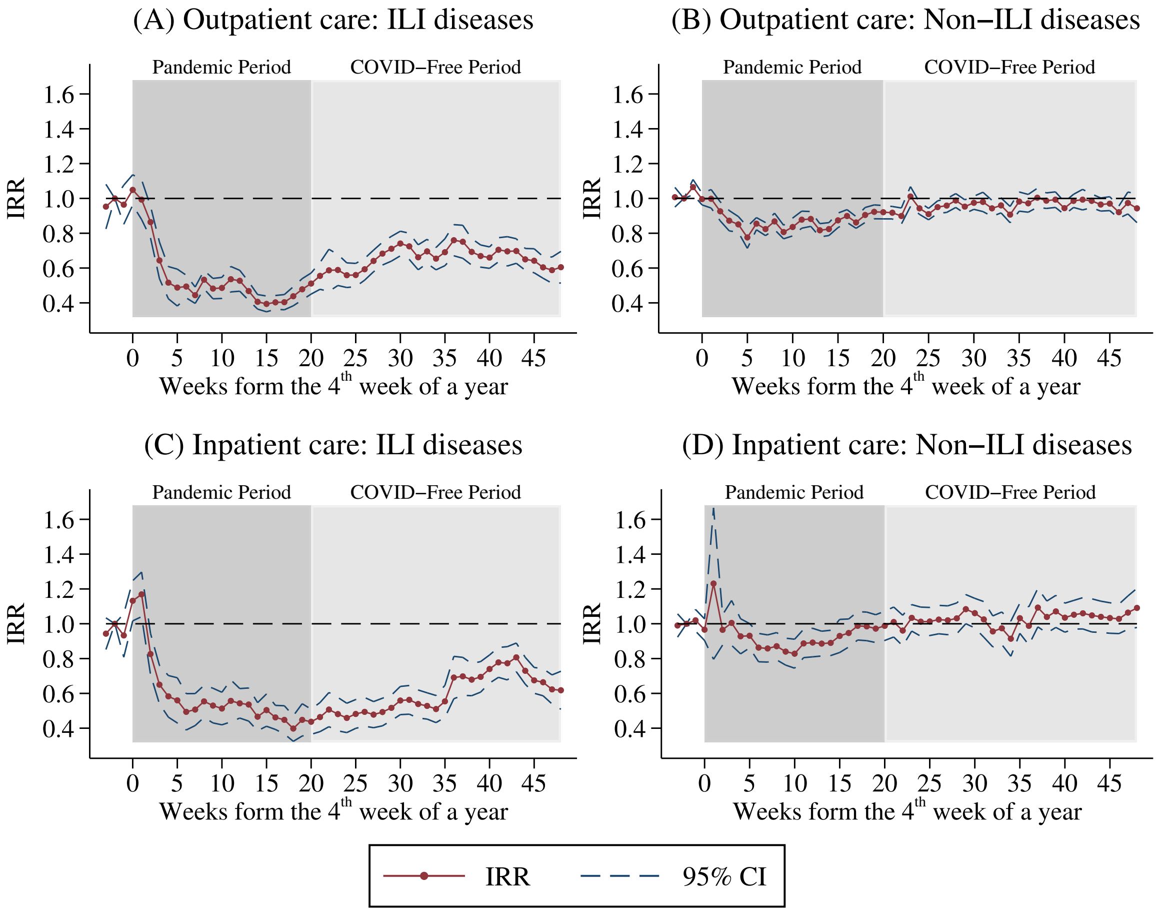

Referee 2's Comment:

7. Table 1: These are mean and standard deviation. I wonder if Taiwan collects population and other demographic characteristics on weekly basis and at a county level. For example, the population pre- to post-outbreak changes. Why?

Authors' Response:

- Thank you for raising this issue. The Taiwanese government collects the population data immediately whenever people (or medical institutions) register or report on newborns, deaths, marriages, immigration, and emigration. The Ministry of Interior (MOI) releases the population statistics on monthly basis. We then merge the monthly population data with the weekly health utilization data. In the revised manuscript, we now discuss this issue and restate the relevant statements as follows:

From the 116th line on page 6:

"To construct our outcome variables—the incidence rate of outpatient visits/inpatient admissions per 100,000 population for specific types of diseases—we divide the number of outpatient visits and inpatient admissions by the population of each corresponding county per year. Population information comes from the population statistics database provided by the Department of Household Registration (DHR), Ministry of Interior (MOI), Taiwan [52]. Population data are measured on a monthly basis. We use the month of the end day of each week to link the weekly day on health utilization with the monthly population data. In our estimated sample, we also include time-varying covariates, such as county-level demographic and weather variables, that may affect health utilization in each county. We acquire demographic data, such as age structure, sex ratio, and educational attainment, from the MOI statistics portal [53]. The above demographic variables are measured on an annual basis. We use the year of the end day of each week to link the weekly day on health utilization with the annual data. We retrieve daily weather information from the Central Weather Bureau's (CWB) observation data inquiry system [54] in order to calculate the weekly average temperature and rainfall for each county."

Referee 2's Comment:

8. Tables 2, 3 and similar tables in the supplements present betas as they come from the software. I would recommend presenting in the main manuscript exponentiated version (which are relative changes) and their confidence intervals. And put the betas as of now in the supplements.

Authors' Response:

- Thank you for your valuable advice. In the revised manuscript, we display all of our estimates in the form of incidence rate ratios (IRRs, the exponentiated coefficient) and report the 95% confidence interval (CI) instead of the raw coefficients from Poisson regression. In addition, we move the original Tables 2 and 3, and Fig 3 (the coefficients version), to S2 Appendix B (labeled Tables B1, B2, and Fig B1, respectively) and report the original robustness checks (Tables B3 to B6) and placebo test (Figs 4 to 6) using an IRR estimate with a 95% CI. Finally, we revise the corresponding description of results in the main text.
